# Cross-sectional serosurvey and associated factors of bluetongue virus antibodies presence in small ruminants of Nepal

**DOI:** 10.1186/1756-0500-7-691

**Published:** 2014-10-06

**Authors:** Tara Nath Gaire, Surendra Karki, Ishwari Prasad Dhakal, Doj Raj Khanal, Nanda Prakash Joshi, Bishwas Sharma, Richard A Bowen

**Affiliations:** Institute of Agriculture and Animal Science, Tribhuvan University, Chitwan, Nepal; Directorate of Animal Health, Tripureshwor, Kathmandu Nepal; Department of Pathobiology, University of Illinois, Urbana-Champaign, IL USA; Animal Health Research Division, Nepal Agricultural Research Council, Lalitpur, Nepal; Department of Large Animal Clinical Science, Michigan State University, East Lansing, MI USA; Department of Biomedical Science, Colorado State University, Fort Collins, CO USA

**Keywords:** Bluetongue virus, Seroprevalence, c-ELISA, Associated factors, Nepal

## Abstract

**Background:**

Bluetongue (BT) is an infectious, insect-borne viral disease primarily affecting sheep and occasionally cattle and goats. In Nepal, BT is an emerging disease of economic importance. The objective of this study was to estimate the seroprevalence of BT virus (BTV) in small ruminants of two eco-zones of Nepal, Hills and Terai, and to identify the factors associated with virus exposure. We conducted a cross-sectional serosurvey from March 2012 through February 2013 by sampling 318 small ruminants (184 sheep and 134 goats) from seven clusters (villages) of selected vulnerable communities of Chitwan (Terai) and Lamjung (Hills) Districts of Nepal.

**Results:**

Of the 318 serum sample tested, 27.9% [95% confidence interval (CI): 23.1- 33.2] were positive for BTV antibodies (25.0% sheep and 31.3% goats). Bivariate analysis indicated a positive association between seroconversion to BTV and flock size, eco-zone, breed, and contact history with cattle. Additionally, in female sheep and goats, a history of abortion was positively associated with seropositivity to BTV. However, the final multivariable model, after controlling for clustering of animals within the villages, identified only history of abortion and breed as the factors significantly associated with BT seropositivity in female sheep and goats. Based on this model, female small ruminants having a history of abortion were more likely to be seropositive compared to those without such history [Odds Ratio (OR) = 46.14 (95% CI: 11.66- 182.5)]. Exotic breeds were more likely to be seropositive compared to indigenous breeds [OR = 9.04 (95% CI: 3.08- 24.46)] while the risk for BTV seropositivity was not significantly different between indigenous and cross breeds.

**Conclusions:**

Our results showed that nearly a quarter of small ruminants in two regions of Nepal were seropositive for BTV, indicating wide exposure of small ruminants to this pathogen. We identified history of abortion and breed as factors significantly associated with the seropositivity of BTV. We recommend that surveillance for BTV infection in Nepal be strengthened and that it would be valuable to enhance the education of farmers about the possible impacts of this disease.

## Background

Bluetongue (BT) is an infectious but non-contagious vector-borne viral disease of both domestic and wild ruminants transmitted by *Culicoides* s*pp* biting midges [1]. Bluetongue virus (BTV) belongs to genus *Orbivirus* in the family *Reoviridae,* and different species of *Culicoides* midges are considered as primary vectors worldwide [2, 3]. BTV is considered endemic in Africa, the Middle East, Australia, and parts of northern hemisphere and Asia [4]. Currently, 26 serotypes of BTV are recognized worldwide [5–7].

Nepal shares borders with India and China, both of which have recognized BT as endemic for several decades. India first reported BT in 1964 in sheep [8] while China first reported the disease in 1979 [9]. In China, antibodies to BTV have been detected in sheep, goats, cattle and buffaloes [9, 10]. There is widespread movement of livestock and people between India and Nepal, whereas in the high Himalayas, Nepali livestock share common pastures with livestock from China. Considering the high seroprevalence in surrounding countries, coupled with transhumance and loose borders with neighboring countries, it would not be surprising that Nepal has BT. Under this scenario, the Government of Nepal has considered BT as one of the priority animal diseases and initiated serosurveillance programs in selected districts. This surveillance program led to the detection of BT infection in sheep in Nepal for the first time in 2008 [11]. Subsequent studies in sheep revealed that 28.4% of the samples from 11 districts were positive for antibodies to BTV [12, 13]. However, in these surveillance programs, only a small number of samples were tested each year and only sheep were tested. Moreover, factors associated with BT seropositivity were not evaluated. Also, baseline data is lacking on seroprevalence in another important small ruminant (goat) in Nepal, resulting in a poor overall understanding of the epidemiology of this disease. The objectives of our study were to evaluate the seroprevalence of BTV in small ruminants (sheep and goats) in two eco-climatic zones of Nepal and to identify the factors associated with BTV seropositivity.

## Results

Serum samples were obtained from 318 small ruminants (184 sheep and 134 goats) from two eco-zones (Terai and Hills). Among sampled small ruminants, 96 were males and 222 were females and the mean and median age of sampled animals were 18.3 months (95% CI: 16.8-19.8 months) and 12 months, respectively. The mean and median total numbers of small ruminants on enrolled farms were 58 and 55, respectively.

Among 318 tested small ruminants, 88 were seropositive by competitive ELISA (cELISA). The apparent seroprevalence, at the individual animal level, was 27.7% (95% CI: 22.9-33.0). The true seroprevalence, after adjusting for sensitivity and specificity of the test, was 27.9% (95% CI: 23.1- 33.3). Out of 88 cELISA-positive samples, 75% (95% CI: 64.6- 83.6) were found to be positive with the agar gel immunodiffusion (AGID) test.

BTV seroprevalence was evaluated based on several additional demographic features of the sampled population, as depicted in the bivariate analyses presented in Table 1, and found to be significantly associated with breed, flock size, history of abortion (in females) and contact history with cattle. In the multivariable analysis using multiple logistic regression, the variables breed (p < 0.0001) and history of abortion (p < 0.0001) remained significant in female small ruminants while none of the variables were significant in males. In female small ruminants, the model containing breed and history of abortion was selected as the final model (Table 2). The odds of being seropositive was 46.14 (95% CI: 11.66- 182.5) times higher in female small ruminants having a history of abortion compared to those not having the history of abortion, and the odds of being seropositive were 9.04 (95% CI: 3.08- 24.46) times higher in exotic breeds of small ruminants compared to indigenous breeds. However, there was no significant difference in the odds of BTV seropositivity between indigenous and cross-bred animals [OR 0.15 (95% CI: 0.02- 1.12)].

**Table 1:** **The results of bivariate analysis of association between bluetongue seroprevalence in goats and sheep and the individual explanatory variables**

Variable and category	Negative animals	Positive animals	OR ^*^ (95% CI ^**^)	P -value
	No. (%)	No. (%)		
Species				
Goats	92 (40.0)	42(47.73)	1	0.212
Sheep	138 (60.0)	46 (52.27)	0.73 (0.44- 1.20)	
Sex				
Female	164 (71.30)	58 (65.90)	1	0.348
Male	66 (28.70)	30 (34.10)	0.79 (0.46- 1.32)	
Age				
<1 yr	100 (43.48)	35 (39.77)	1	0.549
>1 yr	130 (56.52)	53 (60.23)	1.16 (0.71- 1.92)	
Flock size				
Small flocks	55 (23.91)	11 (12.50)	1	0.006
Medium flocks	63 (27.39)	17 (19.32)	1.35 (0.58- 3.13)	
Large flocks	112 (48.70)	60 (68.18)	2.68 (1.35- 5.45)	
Eco-zone				
Hills	111 (48.26)	32 (36.36)	1	0.056
Terai	119 (51.74)	56 (63.64)	1.63 (0.98- 2.71)	
Breed				
Indigenous	156 (67.83)	65 (73.86)	1	0.0001
Cross	66 (28.69)	11 (12.50)	0.4 (0.20- 0.81)	
Exotic	8 (3.48)	12 (13.64)	3.60 (1.41- 9.22)	
Abortion history				
No	145 (88.4)	15 (25.9)	1	<0.0001
Yes	19 (11.6)	43 (74.1)	28.71 (10.25- 46.67)	
Contact history with cattle				
No	193 (83.91)	60 (68.18)	1	0.002
Yes	37 (16.09)	28 (31.82)	2.43 (1.38- 4.30)	

*OR: Odds ratio; **CI: Confidence interval.

**Table 2:** **The final multivariable generalized estimating equations model of bluetongue virus antibodies in female goats and sheep**

Variable and category	OR ^*^ (95% CI**)	P- value
Breed		
Indigenous	1	-
Cross	0.15 (0.02- 1.12)	0.065
Exotic	9.04 (3.08- 24.46)	<0.0001
Abortion history		
No	1	-
Yes	46.14 (11.66- 182.5)	<0.0001

*OR: Odds ratio; **CI: Confidence interval.

## Discussion

We describe a cross-sectional study of BT seroprevalence in small ruminants from two eco-climatic regions (Hills and Terai) of Nepal. There have been few BT seroprevalence studies in sheep in Nepal in the past. In the study reported here, we estimated the seroprevalence of BTV in both goats and sheep and investigated factors associated with BT seropositivity. The major findings from this study were that approximately one quarter of small ruminants tested were positive for antibodies to BTV, and that small ruminants with history of abortion were more than 46 times more likely to be positive compared to small ruminants not having the history of abortion. Additionally, exotic breeds were nearly 9 times more likely to be positive compared to indigenous breeds.

In the present study, the seropositivity of small ruminants was 27.9% (sheep 25% and goat 31.3%). This finding is similar to the findings of Jha and Tamang [12] who detected 28.4% seropositivity in sheep from eleven districts of Nepal. In our study, we found nearly 22% seropositivity in Lamjung (Hills) and 32% seropositivity in Chitwan (Terai). Our observation in Lamjung was higher than reported by Jha and colleagues in 2008 (8.9%) [11] or 2009 (5.3%) [12]. Such discrepancy might be due to the different villages being sampled in different studies or may reflect continued spread of the disease over the last few years. Another possible reason for low seroprevalence in hills may be due to lower temperature, which likely influences the density of vector population. Several studies have shown the distribution patterns of *Culicoides* vectors are related to the spatial variation observed in seroprevalence of BTV [14, 15]. When compared to other Terai districts, seroprevalence in Chitwan (32%) was comparable to that observed in Sunsari (34.3%), however it was lower when compared to Rupandehi (84.2%) [11]. Cattle might have contributed in the transmission of BT to small ruminants in Nepal, as we found that 29% (38/131) of the cattle were seropositive to BTV in the same study area (unpublished result).

As vaccines against BT are not available in Nepal, antibodies detected in small ruminants indicated natural exposure to BTV infection. Vaccination against BTV is not practiced in the neighboring countries India and China also. BTV seropositive animals have been observed from bordering Indian states. For example, 57.6% in sheep in West Bengal [16], 58.8% in sheep in Assam [17] and 54.5% in goats and 13.5% in sheep in Uttar Pradesh [18] were seropositive to BTV indicating widespread exposure to BTV in this area. Nepal shares open borders with India and livestock movement between two countries is very frequent without formal quarantine process, though there is some formal trade through official quarantine routes. Even through official quarantine, animals in incubation period can easily pass through as apparently healthy animals are frequently not quarantined. When disease is present in one country, there is high probability of disease introduction to adjacent countries.

In the present study, sex-wise seroprevalence of BTV was 31.3% in male and 27.2% in female (p > 0.05). Another factor significantly associated with seroprevalence was breed, with exotic breeds showing the highest seroprevalence (60%), followed by indigenous breeds (29.4%), and cross-bred (14.3%). As the number of samples tested from exotic breeds (n = 20) were very less compared to indigenous (n = 221) and cross-bred (n = 77), this might have affected the result. This also indicates that there might be some differences in the local transmission cycle of BT.

We demonstrated significant relationship between seroprevalence and abortion history, with seropositivity in female small ruminants having the history of abortion (74.1%) compared to those that did not (11.6%) (p < 0.0001). This result was not surprising, as BT is known to cause abortion in small ruminants. Our result suggests that BTV may be an important cause of undiagnosed abortion in small ruminants in Nepal. It has been reported previously that BTV can be a significant cause of both abortion and infertility in sheep [18]. In another study, seroprevalence of BTV was higher in dams with a history of abortion [19]. Similarly, in Israel, there was high neonatal morbidity or mortality as well as abortions in goats that were concurrently infected with BTV [20]. Furthermore, BTV is considered as an important cause of abortion in livestock [21, 22]. We did not evaluate the relationship between seroprevalence and abortion history separately for age group. We recommend taking age into account in future analysis as older animals are more likely to have abortions.

A major limitation of the current study was that we did not determine circulating serotype(s) of the BTV. Similarly, our study area was limited to seven clusters (villages) of Lamjung and Chitwan district. A high priority for future studies on BT in Nepal will be to evaluate the circulating serotype(s) of BTV in Nepal as well as temporal variation of seroprevalence by longitudinal studies.

## Conclusions

The present study demonstrated that there was widespread infection of both sheep and goats with BTV, in both eco-zones, Terai and Hills, of Nepal. Of 318 total samples tested, nearly one-fourth animals were positive for antibody against BTV clearly implying that these animals can serve as a potential threat for other small ruminants and cattle in the region and country. History of abortion and breeds were identified as the potential factors associated with BTV seropositivity in female small ruminants. We recommend strengthening of the surveillance system for BT within Nepal and to educate farmers about the management and control of this disease.

## Methods

The study protocol was approved by Institute of Agriculture and Animal Sciences, Tribhuvan University, Nepal. We informed the animal owners about the objectives of our study, obtained their consent before taking samples from animals and communicated test results to the owners. Experienced veterinarians collected the blood samples with no or minimal pain to the animals.

### Study design

**Figure 1:**
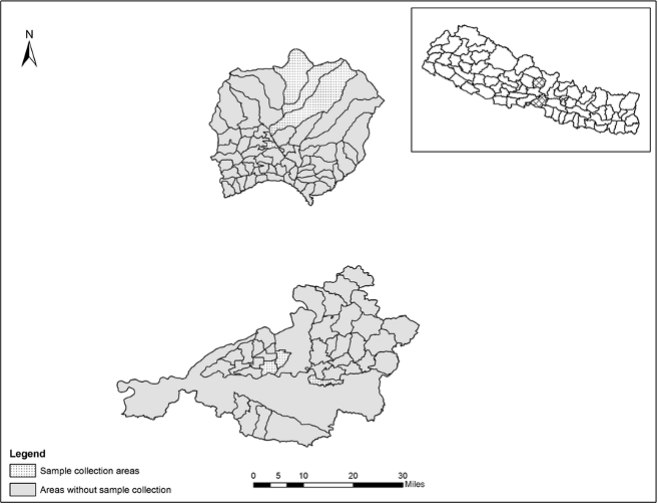
**Map of Lamjung (above) and Chitwan (below) district showing sample collection areas (i.e., village development committees).** The crosses area in the inset shows the location of Lamjung and Chitwan district in Nepal.

We conducted a cross-sectional seroprevalence study in two different eco-zones, Terai and Hills of Nepal (Figure 1) from March 2012 to February 2013, coinciding with the *Culicoides* vector season. Chitwan District (27° 35′ 0″ N, 84° 30′ 0″ E) is part of the Terai lowlands in the Central Nepal (elevation approximately 141 to 1943 meters) and Lamjung District (28° 14′ 0″ N, 82° 25′ 0″ E) is a part of the Mid-hills (elevation between 450 to 8162 meters).

### Sample size and sampling

In total, 318 serum samples from small ruminants (184 samples from in sheep and 134 samples from goats) were collected from 22 farms. Each animal was sampled only once and marked with color for identification after collecting samples. Variables including age, flock size, history of abortion, and history of contact with cattle were recorded based on the information provided by farmers. Sex and breed were recorded at the time of sampling. There was some co-habitation of sheep and goat in Lamjung but not in Chitwan. We also sampled cattle but the detailed results are not included in this analysis.

Five ml samples of blood were collected from a jugular vein from individual animals using sterile evacuated tubes. Serum was separated by centrifugation and transported to National Avian Laboratory, Bharatpur, Chitwan, Nepal, in a cool box containing ice packs at 4°C and stored at -20°C until testing was performed. The samples were tested for the presence of antibodies to BTV using the competitive ELISA test (Veterinary Medical Research and Development (VMRD) Inc., Pullman, Washington, USA). To classify the animal as positive or negative, the cut-off value recommended by the manufacturer was used. Specifically, test samples were considered positive if they produce an optical density less than or equal to 50% of the mean of the negative controls. As per manufacturer, the sensitivity and specificity of this test are 100% and 99%, respectively**.** We also tested the cELISA positive samples using an agar gel immunodiffusion (AGID) test kit, also purchased from VMRD.

### Data management and analysis

Our unit of interest was the individual animal. The result of the c-ELISA test (positive/negative) was the outcome variable. Odds ratio (OR) was used to assess the association between outcome and explanatory variables. Explanatory variables considered were age of the animal, species, breed, sex of the animal, eco-zone, flock size, history of abortion in animals and contact history with cattle. The variable age and flock size were recorded on a continuous scale. However, both of these variables did not have normal distribution as tested by Shapiro-Wilk test (p < 0.0001). Therefore, we classified small ruminants into two age categories: (i) young (less than 1 year old, (ii) adult (greater than 1 year old). We took median age (12 months) as a reference to classify small ruminants in these two categories. For flock size, considering the husbandry practices in Nepal, we categorized flocks into small (less than 50 small ruminants), medium (50–100 small ruminants) and large (more than 100 small ruminants) flocks. The apparent seroprevalence was calculated by dividing the number of positive samples by the total number of samples tested. The true prevalence was then estimated using the formula: True prevalence = (Apparent prevalence + Specificity-1)/(Sensitivity + Specificity-1)) [23].

For statistical analyses, we used SAS 9.3 software (SAS Institute Inc., North Carolina, USA). Bivariate associations between the outcome and individual explanatory variables were assessed using the Pearson’s Chi-square test. Liberal cut-off of 20% was used for variable selection to be included in the multivariable analysis. Multivariable analysis was performed using the generalized estimating equation (GEE) approach (the PROC GENMOD command in SAS) to account for the clustering effect at the village (clusters) level separately for males and females as history of abortion was not applicable to male population. Final multivariable model was selected using the backward variable selection approach. We assessed the two-way interactions between individual explanatory variables. P-values less than 0.05 were considered significant.
